# Cyberbullying and Cyberhate as Two Interlinked Instances of Cyber-Aggression in Adolescence: A Systematic Review

**DOI:** 10.3389/fpsyg.2022.909299

**Published:** 2022-05-27

**Authors:** Giovanni Fulantelli, Davide Taibi, Lidia Scifo, Veronica Schwarze, Sabrina C. Eimler

**Affiliations:** ^1^Institute for Educational Technology, National Research Council of Italy, Palermo, Italy; ^2^Institute of Computer Science, Institute of Positive Computing, University of Applied Sciences Ruhr West, Bottrop, Germany

**Keywords:** cyberbullying, cyberhate, cyber-aggression, adolescents, social media, social network sites

## Abstract

In this paper we present the results of a systematic review aimed at investigating what the literature reports on cyberbullying and cyberhate, whether and to what extent the connection between the two phenomena is made explicit, and whether it is possible to identify overlapping factors in the description of the phenomena. Specifically, for each of the 24 selected papers, we have identified the predictors of cyberbullying behaviors and the consequences of cyberbullying acts on the victims; the same analysis has been carried out with reference to cyberhate. Then, by comparing what emerged from the literature on cyberbullying with what emerged from the literature on cyberhate, we verify to what extent the two phenomena overlap in terms of predictors and consequences. Results show that the cyberhate issue related to adolescents is less investigated than cyberbullying, and most of the papers focusing on one of them do not refer to the other. Nevertheless, by comparing the predictors and outcomes of cyberbullying and cyberhate as reported in the literature, an overlap between the two concepts emerges, with reference to: the parent-child relationship to reduce the risk of cyber-aggression; the link between sexuality and cyber-attacks; the protective role of the families and of good quality friendship relationships; the impact of cyberbullying and cyberhate on adolescents' individuals' well-being and emotions; meaningful analogies between the coping strategies put in practice by victims of cyberbullying and cyberhate. We argue that the results of this review can stimulate a holistic approach for future studies on cyberbullying and cyberhate where the two phenomena are analyzed as two interlinked instances of cyber-aggression. Similarly, prevention and intervention programs on a responsible and safe use of social media should refer to both cyberbullying and cyberhate issues, as they share many predictors as well as consequences on adolescents' wellbeing, thus making it diminishing to afford them separately.

**Systematic Review Registration:**

http://www.crd.york.ac.uk/PROSPERO, identifier: CRD42021239461.

## Introduction

Social media have become online environments in which face-to-face activities of everyday life are transferred in the network mediated world with a wider audience (potentially millions of users) and no time constraints (they are open 24 h a day). According to the Digital 2020 July Global Statshot report^1^, July 2020 can be considered a milestone in the history of the internet, since for the first time more than half of the world's total population was using social media, with a total number of 3.96 billion active social media users (Kemp, 2020). Due to the coronavirus pandemic lockdowns, this number increased up to 4.33 billion active social media users in April 2021 (55.1% of world population, with an annual increase close to 14%) (Kemp, 2021a)^2^.

The share of adolescents contributing to these numbers is impressive: 90% of US teens aged 13-17 years use social media (AACAP, 2018)^3^; a similar percentage concerns Europe, where 87% of people aged 16 to 24 years use social networks, ranging from 79% in Italy up to 97% in Denmark (Eurostat, 2020,^4^; research from GWI shows that 99.6% of South-East Asian internet users aged 16–24 years use social media (Kemp, 2021b).

According to these statistics, adolescents are among the most frequent users of social media. Following Shapiro and Margolin (2014), the main motivations for adolescents using social media are “to stay in touch with friends, make plans, get to know people better, and present oneself to others”.

Despite these perceived or real benefits, social media can become a place in which antisocial behaviors such as bullying, harassment and hate speech, proliferate and evolve leveraging the peculiarity of the online world (ElSherief et al., 2018). As a consequence, participation in social media exposes adolescents more to the risks associated with their use. In particular, several studies have pointed out the relationship between social media use and cyber-violence, broadly defined as violent acts perpetrated through the social media (Peterson and Densley, 2017; Backe et al., 2018; Nagle, 2018), and how adolescents can become victims or perpetrators of aggressive behaviors (Chisholm, 2006; O'Keeffe et al., 2011; Peterson and Densley, 2017). Furthermore, some authors have highlighted the difficulties in classifying and defining the spectrum and diversity of online violent behaviors, their specificity compared to similar offline behaviors, and the limits that result from a lack of clear definitions of online violent behaviors (Grigg, 2010; Pyzalski, 2012; Peterson and Densley, 2017).

It is precisely from the difficulties in defining the concept of cyberbullying that Grigg (2010) comes to the conclusion that it is necessary to move to a concept at a higher level of abstraction that includes all the online behaviors characterized by a high level of aggression, thus introducing the concept of cyber-aggression: “The study examined current definitions and concepts of cyberbullying and how these differ in its findings; and considered different ways to foster positive online behavior for the context of practitioners. The concept of cyber-aggression is used to describe a wide range of behaviors other than cyberbullying. The findings indicate that there is a need to include a broader definition in line with the current trend of a range of behaviors that are common with internet and mobile phone usage” (p. 143). Following Grigg's idea of cyber-aggression, Corcoran et al. (2015) argue that, in order to overcome the problems related to the variations across definitions of cyberbullying, it is necessary to consider the broader issue of cyber-aggression.

In addition to cyberbullying, cyberhate is another important example of online aggressive behavior which is more and more involving young victims and perpetrators. This is related to two main factors: firstly, the amount of online hate material (such as hateful messages and, more in general, content that harm the reputation of or instigate violence against groups or individual as member of groups) is rapidly increasing, and the risk for adolescents to be exposed to hateful online material is increasing accordingly (Hawdon et al., 2019; Harriman et al., 2020); secondly, adolescents are becoming one of the preferred target for online recruitment by organized hate groups and individuals (Smith, 2009; Costello et al., 2020). Similar to cyberbullying, several authors consider cyberhate as a subset of cyber-aggression (Mardianto et al., 2019; Tennakoon, 2021; Bedrosova et al., 2022).

As mentioned before, adolescents are particularly vulnerable to cyber-aggression, not only because of the time spent online: US statistics in 2018 showed that nearly all teens aged 13–17 years (95%) have access to a smartphone and 45% of them reported that they were online “almost constantly” (Anderson and Jiang, 2018); adolescents are at high risk also because most of them have fewer psychological tools than the majority of adults to defend themselves against cyber-aggression, such as resilience, competence, literacy, critical thinking, experiences.

Cyberbullying and cyberhate play a dramatic role in the relationships between adolescents' well-being and use of social media. In fact, cyberbullying is the most frequent form of cyber-aggression involving adolescents, while cyberhate is the form of cyber-aggression that is spreading most rapidly among young people. Furthermore, the two phenomena are not totally distinct, rather there are some overlaps between them (Goerzig et al., 2019; Bedrosova et al., 2022). However, to the best of our knowledge, there is no approach addressing cyberbullying and cyberhate as two distinct but interlinked phenomena which makes it hard to evaluate them in empirical research settings. As a consequence, analyzing the two phenomena not in a separate manner, but on the contrary assuming that there may be important links between them, can help to develop models for the identification of predictor variables, to broaden the assessments of the possible impacts they have on the lives of adolescents, and to put in practice more effective and efficient prevention strategies. This approach can offer a concrete model which would be of interest to academia to explain how theoretical models can help to derive practical interventions to limit the spread of these phenomena. In the long term, the review can support practitioners in the school contexts in developing intervention measures aimed at avoiding toxic dynamics on the internet.

Accordingly, in this paper we present the results of a systematic review aimed at investigating what the literature reports on cyberbullying and cyberhate, whether and to what extent the connection between the two phenomena is made explicit, and whether it is possible to identify what exactly the overlapping factors are. Specifically, for each of the analyzed papers, we have identified the predictors of cyberbullying behaviors and the consequences of cyberbullying acts on the victims; the same analysis has been carried out with reference to cyberhate. Then, by comparing what emerged from the literature on cyberbullying with what emerged from the literature on cyberhate, we verify to what extent the two phenomena—as reported in the literature—overlap in terms of predictors and consequences.

### Cyberbullying and Cyberhate as Two Interconnected Phenomena

In order to reflect on the relationship between cyberbullying and cyberhate in terms of differences and similarities, we focus on the distinguishing characteristics of each of them, being aware that the definition of the two concepts is not an objective of this review. In both cases, we focus on the comparisons between these two forms of cyber-aggression and their equivalent forms in face-to-face contexts: bullying and hate speech.

Cyberbullying does not simply refer to the transition from 'traditional' bullying in face-to-face contexts to bullying in online contexts, where, according to the Centers for Disease Control and Prevention, bullying is defined as “*any unwanted aggressive behavior(s) by another youth or group of youths who are not siblings or current dating partners that involves an observed or perceived power imbalance and is repeated multiple times or is highly likely to be repeated*.”^5^ In fact, the characteristics of social media lead to a re-interpretation of the concepts of aggression, repetition and the imbalance of power (Whittaker and Kowalski, 2015). Firstly, the characteristics of physical aggression related to vocal tone and facial expression assume a different value in the virtual world, in which other shapes of aggression come into the scene through hate speech or online harassment, humiliation or exclusion. Concerning repetition, in an online environment a harassing statement can be potentially viewed, “liked” and shared by other users multiple times, therefore the repetition of the act over time is not more crucial, only one shared content humiliating a victim could have a destructive effect on the victim's self-esteem. Finally, power imbalance is difficult to detect in a virtual environment in which power can be expressed in a multitude of ways. For example, users with a high level of digital knowledge can conduct cyberattacks by using sophisticated tools, so that power imbalance might also reflect differences in technological expertise (Whittaker and Kowalski, 2015).

Other characteristics of the virtual environment affect the proliferation of cyberbullying and differentiate cyberbullying from traditional bullying. In the virtual environment cyberbullies can use anonymous accounts to attack their victims. There is another relevant difference between cyberbullying and bullying as stated by Englander (2017) who state that cyberbullying is connected to the widespread use of digital devices, thus leading cyberbullying to happen mainly outside of school, whereas traditional bullying most often happens in school.

Moving to the cyberhate concept, there are not universally accepted definitions of hate speech and cyberhate (MacAvaney et al., 2019). Specifically, to hate speech, differences amongst the definitions concern several aspects. Firstly, the authors and the spreaders of the hate messages can be individuals, organized groups or a combination of individuals and organized groups (Blaya and Audrin, 2019). Then, the target or victim is one of the most variable concepts in the definitions of hate speech, and it strongly reflects the differences in the contexts of use and in the historical period of definition. Some of the definitions consider only specific groups, such as the one proposed by the Council of Europe in the Additional Protocol to the Convention on Cybercriminality (Council of Europe, 2003) that states that individuals or communities become target of attacks because of their “race, color, descent or national or ethnic origin, as well as religion if used as a pretext for any of these factors”; the constraints posed to the potential target groups of hate speech is due to the origin of this document, conceived as a normative instrument to contrast racism and xenophobia. On the other end of the spectrum of definitions, the Center for Equal Opportunities and Opposition to Racism in Brussels lists sex, sexual orientation or political or religious beliefs, in addition to skin color, supposed race, ethnic origin, as reasons to unleash the haters.

A further distinction concerns the purpose of haters. The Council of Europe states that hate speech aims at advocating, promoting or inciting hatred, discrimination or violence (Council of Europe, 2003). Blaya and Audrin (2019) clarify that the purpose of haters is to attract new members toward their ideals, thus building and strengthening group identity to counter and reject others' collective identity. The mechanisms of propaganda, insulting and discrimination are therefore central to the hate speech definitions (Council of Europe, 2003; Anti-Defamation League, 2010; Blaya and Audrin, 2019). One of the definition that better resumes and mediates the different perspectives to hate speech is the one proposed in 1997 by the Council of Europe, which refer to hate speech as “all forms of expression which spread, incite, promote or justify racial hatred, xenophobia, anti-Semitism or other forms of hatred based on intolerance, including intolerance expressed by aggressive nationalism and ethnocentrism, discrimination and hostility toward minorities, migrants and people of immigrant origin” (Council of Europe, 1997).

Moving to the differences between hate speech and online hate speech or cyberhate, the most evident difference is the media used for disseminating hate content and messages. The Council of Europe (2003) provides a comprehensive view of hate speech material, which includes “any written material, any image or any other representation of ideas or theories”. Drawing on this definition, Blaya and Audrin (2019) adopt the term “cyberhate” to refer to all hateful online forms of expression (text, images, videos, pictures, graphic representations) to generate hatred against people and communities. Cyberhate is based on the spreading of hateful material through electronic communication technologies (e.g., websites, social media, blogs, online games, instant messaging services, e-mail). As for cyberbullying, the use of Internet-based communication media amplifies the effect of cyberhate, and exacerbates the negative consequences of hate speech.

Differences between cyberbullying and cyberhate are a direct consequence of the definitions provided so far. Firstly, as already mentioned, the final purpose of cyberhate is to promote or incite hatred, discrimination or violence against a community or group in order to disaggregate social cohesion and mine democracy; instead, the final aim of a cyberbullying is to harm an individual.

Bullying is known with its repetitive act to the same individual, unlike hate speech which is more general and not necessarily intended to hurt a specific individual (Al-Hassan and Al-Dossari, 2019). As Chetty and Alathur (2018) claim, hate speech may harm the victims directly or indirectly. Direct hate speech is similar to bullying, since the victims are injured immediately by hate speech content. However, in an indirect hate speech, the harm perpetrated by the original hater is only a part of the final goal, since the hater incites other people or organized groups to attack the victims, and a delayed harm is perpetrated by the latters, not by an original actor. In a typical racist hate speech scenario, hateful content on racism in (real or online) public settings might motivate other people to initiate harassment, intimidation, violence against ethnical minorities (Seglow, 2016). This introduces a second difference: one of the aims of the cyberhaters is to involve as many people as possible as active agents in the attack; this is not a priority for the perpetrator of cyberbullying, who is normally an individual or a small group of peers. Consequently, the online services used by the two categories of haters are different, even if some overlaps exist.

Strictly related to the previous one, another difference is that cyberhate targets communities more than individuals, while cyberbullying victims are individuals, usually young individuals in the setting of a particular community, like a school (López and López, 2017). Following Blaya and Audrin (2019), cyberhate can also harm individuals and affect them emotionally, but the main negative consequences are on whole communities. Another difference is that the perpetrator of cyberbullying usually personally knows his/her victim, which is often unknown to most of the cyberhaters.

Finally, the idea of victimization changes accordingly: specifically to cyberhate, Machackova et al. (2020) distinguish between cyberhate exposure and cyberhate victimization, and they define the former as “the experience of encountering hateful content online but not necessarily feeling victimized by it”. This distinction is not necessary in cyberbullying, since it is always possible to identify a victim.

Despite the several differences between the concepts of cyberbullying and cyberhate, they share important characteristics that lead to a partial overlap between the two concepts and promote the study of similar solutions. As mentioned before, in both cases the use of Internet-based technologies amplifies the consequences of the attacks, both in terms of geographical space and persistence over time. Secondly, the lack of face-to-face contact between the perpetrator and the victim, both in cyberbullying and in cyberhate, makes online forms of expression (text, images, videos, pictures, graphic representations) the common language used by the perpetrators. Then, school-aged children, adolescents and young people are particularly exposed to cyberbullying and cyberhate, both as victims and perpetrators: Li et colleagues (2021) have illustrated that cyberbullying perpetration among school-aged children is a transnational phenomenon; likewise, organized hate groups specifically target adolescents as new recruits (Lee and Leets, 2002; Gerstenfeld et al., 2003), so that they are particularly vulnerable to online media activist groups due to their presence in social media, and the particular stage of their development who makes them sensitive to feelings of personal commitment, of social utility and of belonging they can provide (Atran and Ginges, 2015); finally, the concept of victimization is central to both, cyberbullying and cyberhate, in particular by taking into account that a large number of victims and perpetrators are adolescents.

In addition to these general factors that characterize the nature of the two phenomena, the aim of this review is, as anticipated in the introduction, to analyze in more detail the individual factors that—based on the results of the empirical studies presented in the selected papers—allow us to understand the level of overlap between cyberbullying and cyberhate. This will make it possible to better target interventions aimed at preventing the phenomena of cyber-aggression committed by or directed against adolescents.

## Materials and Methods

The PRISMA method was followed for the review methodology and data extraction (Liberati et al., 2009; Moher et al., 2009; Page et al., 2021). A protocol for this review was registered on PROSPERO in April 2021 (PROSPERO registration number CRD42021239461. The registration number is available at http://www.crd.york.ac.uk/PROSPERO).

### Participants and Procedure

To identify the literature, the following databases were searched: PsycInfo, Scopus, PubMed, APA PsycArticles, EBSCO. The search for electronic literature databases was dated from January 2021 to February 2021. In each database, the following terms were searched: social media, adolescents, boys, girls, young adults, teenagers, cyberbullying, cybermobbing, online hate speech, cyberhate, cyber victimization. The search strategy was carried out combining these keywords with boolean operators as AND, OR, NOT. Of the selected empirical studies, the chronological age of research participants between 10 and 24 years were considered. Moreover, the broad age range of research participants was a forced choice because several studies included both adolescents and young adults. The selection of the studies was a process of evaluation of synonyms and related keywords, as the scientific literature concerning the topic of the review is vast and articulated.

In order to ensure that no relevant studies were missed, additional studies were identified by hand-searching the reference lists of reviews and research papers. Missing papers were requested from study authors by email.

The papers/records included in the review and analyzed are 24.

### Inclusion and Exclusion Criteria

All the records were independently screened by four review authors to identify studies that potentially met the inclusion and exclusion criteria as outlined below.

The following inclusion criteria were adopted:

- studies described different types of cyberbullying and cyberhate in social media
- quantitative study and empirical papers published between 2000 and 2021
- studies were published in the English language
- empirical studies, experimental and quasi-experimental design
- peer-reviewed studies
- people aged between the ages of 10 and 24.

In the exclusion criteria, duplicates and irrelevant records have been eliminated.

In particular, the following exclusion criteria were adopted:

- qualitative studies
- studies were published before 2000
- studies were not published in the English language
- cross-sectional design, single case
- studies were not peer-reviewed
- gray literature, e.g., dissertations, conference abstracts, research reports, chapter(s) from a book, Ph.D. theses, reports on ID guidelines.

### Data Analysis

**Figure 1:**
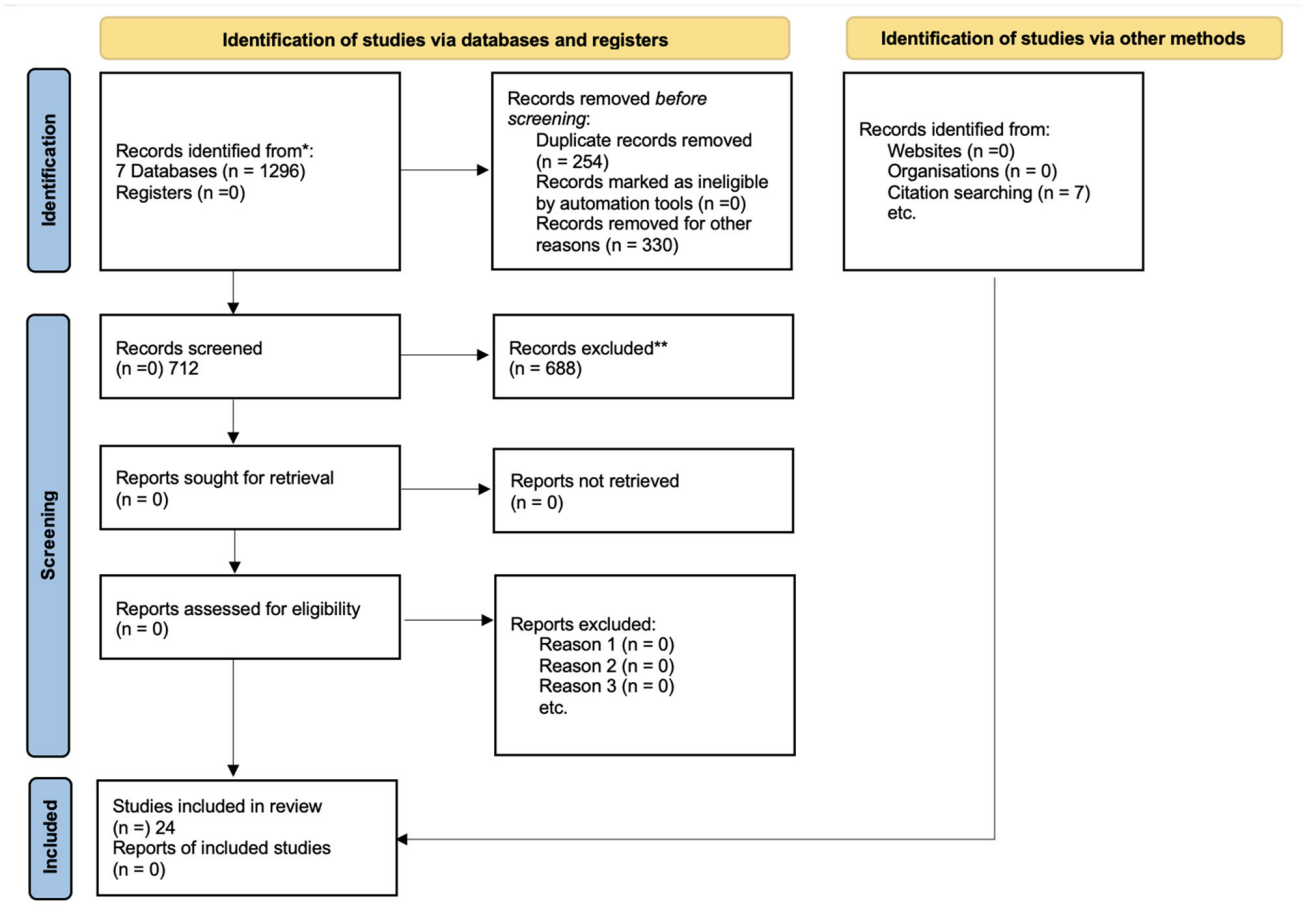
PRISMA flow chart of the search and selection process.

The initial database search identified a total of 1296 records, after a careful selection, according to the PRISMA checklist and the inclusion and exclusion criteria, 24 papers were analyzed for the data extraction. The flow diagram following the models of Page et al. (2021) is included as Figure 1.

Papers were firstly reviewed through the title and abstract to determine if they could be included or excluded. The final papers were organized for the data extraction according to the following variables: digital object ID, article title, abstract, journal title, journal year, authors, social media as the subject of investigation, which kind of cyberbullying/cyberhate, content of the studies, method and procedure participants, age participants, sex, stage of life, country, aim of the study, result.

The synthesis of the main variables considered essential to the topic of the systematic review process is reported in Table 1.

**Table 1:** Main characteristics of the papers included in the review.

**Author/s year**	**Characteristics of the sample**	**Subject of investigation**	**Hate speech, (Cyber)bullying**	**Social media**	**Main findings**	**Paper key word**
Mitchell et al. (2011)	2.051 adolescents age range 10–17 years	To examine rates of victimization, and the association between online and offline victimization. In particular, the symptoms of trauma and delinquency among adolescents were analyzed	Sexual victimizations, and psychological and emotional abuse	Not specified	Results show that many victims are at risk because they have very complex previous emotional experiences. Offline victimization experiences are associated with online victimization	Internet; Victimization; Adolescents; Trauma; Delinquency; Life adversity
Bossler et al. (2012)	434 adolescents students in a Kentucky middle and high school	To explore online harassment experiences. In particular, it was examined whether routine computer activities including use of computers increase the risk of victimization	Online harassment,bullying	MySpace, Facebook	Results show that having a social networking site and sharing personal information on these online platforms seemed to make harassment more likely than using tools such as email or instant messaging	Online harassment, routine activities theory, bullying
Ybarra et al. (2011)	1,588 adolescents age range 10–15 years	To examine technology-mediated exposures (e.g., hate sites, death sites) and experiences (e.g., bullying) and how they are associated with psychosocial challenges (e.g., violent behavior, depressive symptomatology)	Cyberbullying, Internet harassment unwanted sexual solicitation, unwanted sexual experiences	Text-Messaging, Violent Web sites, Hate sites, Design-based, War, death, and “terrorism” sites Design-based, Cartoons sites Design-based, Violent x-rated (“adult”) sites	Results show that while youth move online, victimization rates increase. Almost all violent experiences and exposures online are caused by the use intensity and frequency of the Internet and text messaging	Youth, violent media, Internet harassment, unwanted sexual solicitation, unwanted sexual experiences, cyberbullying
Oksanen et al. (2014)	723 Adolescents age range 15–18 years	To analyze data collected from a sample of Finnish Facebook and YouTube adolescent users. This research investigated the extent of exposure to and victimization by online hate material among young social media users	Hate material, victimization	Facebook and YouTube	Results show that exposure to hate material was associated with high online activity, poor attachment to family and physical offline victimization. Online hate material primarily focused on sexual orientation, physical appearance, and ethnicity	Victimization, internet, adolescents, youth, hate material
Pauwels and Schils (2016)	6,020 Adolescents age range 16–24 years	To apply Social Learning theory to the explanation of political violence, focusing on exposure to extremist content through new social media and Facebook	Youth delinquency, youth delinquency, differential association exposure	Facebook, NSM	Results show that the most violent effects of new social media and Facebook are found for those measures where individuals actively seek out extremist content on the Internet. It is necessary to check background	Nonstate actors, Radicalization, Terrorism / counterterrorism, Violent extremism
					Variables such as personality characteristics, moral values and peer influences	
Räsänen et al. (2016)	723 adolescents age range 15–18 years	To examine whether the risk of online hate victimization is more likely when youth visited online sites containing potentially harmful content	Victimization, online hate social media	Facebook	Results show that being involved in the production of hate material and in researching such content one puts young people in danger	Social media; routine activity theory; victimization; online hate
Baldry et al. (2019)	5,058 adolescents age range 11–18 years	To investigate post-traumatic stress symptoms affecting the involvement in school bullying and cyberbullying of boys and girls according to the different bullying roles	Cyber bullying School bullying Cybervictimization	Not specified	Results show that school and cyberbullying are risk factors for development of post-traumatic stress symptoms differently affecting adolescents according to their role	School children, post-traumatic stress disorder, schools, symptoms, abuse, adolescents, aggressive behavior, boys, girls, human behavior, human diseases, risk factors, risk groups, students, children
Longobardi et al. (2020)	345 adolescents age range 11–16 years	To analyze the association between Instagram popularity and subjective happiness and evaluate the relationship between roles of cyber victimization and social media addiction	Cyber victimization, Social media addiction	Instagram	Results show that social media use and cyber victimization were positively correlated, and both showed a negative correlation with perceived subjective happiness	Cyber victimization, Instagram, Peer exclusion, social media addiction, Happiness, Well-being, Adolescents
Blaya et al. (2020)	1,900 students age range 12–20 years	To examine the association between school bullying and cyberhate victimization and perpetration	Cyberhate, bullying, victimization and perpetration	Not specified	Results show that bullying and cyberhate are a common experience for many young people. In particular, the overlap between bullying and cyberhate and between traditional bullying and cyberbullying is evident	Cyberhate, Young people, Victimization, Involvement, School bullying, Overlap
Wachs et al. (2020)	1,480 adolescents age range 12–1ears	To investigate adolescents' coping strategies for cyberhate, while considering differences in adolescents' sex, age, socioeconomic status (SES), and victim status	Cyberhate, coping strategies, adolescents, cyberbullying	Not specified	Results show that different coping strategies are used by adolescents, with differences depending on sex, age, socioeconomic status, and victim status	Cyberhate Coping strategies Cybervictimization Hate speech Cyber discrimination
Ang and Goh (2010)	396 adolescents age range 12–18 years	To examine the association between affective empathy, cognitive empathy, and gender on cyberbullying among adolescents	Cyberbullying among adolescent (for an example, hurting someone by sending them rude text messages)	Not specified	Results show that both boys and girls who had low cognitive empathy had higher scores on cyberbullying than those who had high cognitive empathy	Cyberbullying, Affective empathy, Cognitive empathy, Gender
Barlett et al. (2019)	3,079 youth participants average age 13.12 years (Wave 1) 1,957 youth participants (Wave 2) 909 youth participants (Wave 3)	To testing (a) the longitudinal stability in positive cyberbullying attitudes (CA), (b) whether any change in positive CA over time predict subsequent cyberbullying perpetration, and (c) the cross-lagged relations between positive attitudes toward CA and behavior over time	Positive cyberbullying attitudes, cyberbullying perpetration. (Relation between attitude and behavior)	Not specified	Results show a modest stability in positive CA and perpetration over time. Latent class analysis classified participants into either stable high attitudes, stable low attitudes, increasing attitudes, or decreasing attitudes	Cyberbully, cyberbullying attitudes
Gradinger et al. (2009)	761 adolescents age rage 14–19 years	To analyzed whether students in the world both traditional and cyber belonging to groups of bullies or victims and bully and victims differed regarding adjustment	Traditional bullying, cyberbullying, traditional victimization, and cybervictimization	Mobile phones and computers (Not further specified)	Results show that the highest risks for poor adjustment were observed in students who were identified as combined bully-victims (traditional and cyber). In addition, gender differences were examined	Cyberbullying, cybervictimization, adjustment, aggression, configural frequency analysis
Schneider et al. (2012)	20,406 students 9th through 12th grade	To examine the prevalence of cyberbullying and school bullying victimization and their associations with psychological distress	Bullying victimization and psychological distress, including depressive symptoms, self-injury, and suicidality	Not specified	Results show that victimization was higher among no heterosexually identified youths. Victims report lower school performance and school attachment. Distress was highest among victims of both cyberbullying and school bullying	Adolescent, Bullying, Psychological epidemiology, Stress, Psychological etiology
Low and Espelage (2013)	1,023 early adolescents age range 10–15 years	To understanding the role of maladaptive family social dynamics on cyber-bullying and nonphysical bullying (i.e., verbal and relational) involvement through individual risk and protective factors	Cyber-bullying and nonphysical bullying (i.e., verbal and relational)	Not specified	Findings validate the importance of familial socialization. Cyber-bullying shows a significant overlap with nonphysical bullying, in particular, nonphysical bullying levels were associated with both higher family	Bullying, cyberbullying, longitudinal predictors, race, gender
					Violence and lower parental monitoring	
Mehari and Farrell (2018)	677 adolescents age range 11–15 years	To assess whether the dimensional model that cyberbullying that fits into a framework of adolescent aggression considered both form (overt or relational) and media (in-person or electronic) best fit the data	Form (overt or relational) and media (in-person or electronic) of aggression	Not specified	Results show that cyberbullying is a new form of aggression, a counterpart to overt and relational aggression, and this conceptualization fits the data quite well	Aggression, cyberbullying, adolescence, measurement of aggression, electronic media
Mishna et al. (2010)	2,186 students grades 6, 7, 10, and 11	To examine technology use, cyberbullying behaviors, and the psychosocial impact of bullying and being bullied, among a large sample of middle and high school students in a large urban center	Cyberbullying and technology use	Not specified	Results show that bullying was perpetrated by and toward friends and that bullies do not often disclose that they have been bullied. After the assault, the online bully reported feeling angry, sad, and depressed	Adolescents, Canada, cyber bullying, victimization, gender, ethnicity
Ortega-Ruiz et al. (2009)	1,671adolescents age range 12–17 years	To examine the emotional impact caused to victims of traditional bullying or cyberbullying through technologies such as cell phones and the Internet	Direct bullying, indirect bullying, bullying inflicted *via* mobile phone, bullying inflicted *via* internet	Mobile phones and internet (no further specification)	Results show that traditional bullying affected more youth than cyberbullying. Cyberbullying produces emotional profiles like traditional bullying. The most common emotional response is anger and other negative emotions	Bullying, cyberbullying, emotions, victimization, adolescents
Patchin and Hinduja (2010)	1,963 students age range 10–16 years	Examines the relationship between cyberbullying and their level of self-esteem in the experience of middle school students	Cyberbullying	Email, MySpce or other (not further specified) web page	Results show that students who were both victims and perpetrators in cyberbullying had significantly lower self-esteem than those who had little or no experience with cyberbullying	Aggressive behavior, behavior, human behavior, programmes, psychological factors, school buildings, school kids, schoolchildren, teenagers, United States of America
Schultze-Krumbholz and Scheithauer (2009)	71 students grades 7, 8, and 10 average age 14.05 years	To identify characteristics of cyberbullies and cybervictims to be considered as potential risk and/or protective factors in a future study with a larger sample of students. Specifically, the	Cyberbullying and cybervictimization	Email, mobile phones and internet in general	Results show that higher frequency of cyberbullying compared with traditional bullying, and an overlap between cyberbullying and cybervictimization. Also, cyberbullies and cybervictims	Cyberbullying, social behavioral correlates, cybervictimization, empathy, frequency analysis
		Research aims to assess the quality of several measurement instruments			Showed less empathy and higher relational aggression	
Ševčíková and Šmahel (2009)	Different age groups, including 223, respectively, 224 younger adolescents (age range 12–15 years) and 248, respectively, 249 older adolescents (age range 16– 19 years)	To explore the frequency of online aggressive acts (as victim and aggressor)	Cyberbullying and aggressive behavior	Not specified	Adolescents (12–19 years old) were more often the target of aggressive behavior than older respondents	Harassment cyberbullying Internet Czech Republic
Waasdorp et al. (2018)	26,494 high school youth and 16,749 middle school youth	To analyze if weight status exacerbates the association between victimization and internalizing symptoms in bullied obese youth	Association between different forms of victimization, weight status, and adjustment	Not specified	Results highlight an increased risk of psychosocial adjustment problems among obese and overweight youth who are frequent victims of bullying. The odds of experiencing cyber victimization were higher than the odds of experiencing other forms of victimization	Internalization, Obesity, Overweight, Victimization, Bullying, At Risk Populations, Peers, Symptoms, Test Construction, Adolescent Characteristics, Internalizing Symptoms, Adolescence (13-17 yrs), Male, Female
Yang et al. (2020)	16,237 adolescents 6th−12th grade	To explore the relationship between cyberbullying victimization (CBV), student emotional engagement, and cognitive-behavioral engagement at both the student and school level	Traditional bullying victimization, cyberbullying victimization	Not specified	The most relevant findings suggest that CBV had a small but significant positive association with emotional engagement and a small but significant negative association with cognitive-behavioral engagement	Bullying victimization, cyberbullying victimization, school climate, student engagement
Zaborskis et al. (2018)	3,814 adolescents mean age 15.67 years	To analyze the prevalence of bullying and cyberbullying and their association with suicidal behavior among school-aged children in Israel, Lithuania, and Luxembourg	Cyberbullying and suicidality	Not specified	Results show that cyberbullying is a strong predictor of adolescent suicidality	Adolescents, bullying, cyberbullying, suicidality, associations

To systematically report and compare the heterogeneous findings of the articles included, a coding scheme was created, with categories derived from the reported results. Since some papers contained a large number of different worth reporting findings, the results were not exclusively assigned to one category, but are presented in the context of different dimensions. Given that the focus of this review was on cyberbullying and cyberhate as two interlinked instances of cyber-aggression, both phenomena contributed to the main distinction of the results, with 19 papers referring to cyberbullying and 5 papers referring to cyberhate. Next, the results were categorized according to whether they related to the predictors or the impact of cyberbullying or cyberhate. Thus, a 2 × 2 matrix was created, which served as a grid for the analysis of the results. The grid was further divided into subcategories as follows: as predictors, we considered (1) socio-demographic variables (e.g., age/school grade level, gender, race/ethnic background), (2) individual and contextual factors (e.g., empathy, sexuality, appearance/overweight, school performance, relationships with friends and family) and (3) the overlap between traditional and cyberbullying as well as the overlap of cyberhate with other forms of aggressive behavior. Similarly, the effects of both phenomena were contrasted and considered categories such as effects on health and well-being (e.g., psychological distress, depressive symptoms, somatic symptoms, suicidal) and coping strategies.

## Results

### Predictors of Cyberbullying

#### Socio-Demographic Variables

Numerous studies investigated the potential impact of various socio-demographic variables on cyberbullying behaviors. Age seems to play a substantial role in the prevalence of these behaviors. For example, Ybarra et al. (2011) found evidence for increasing age being predictive for exposure to and experience of online harassment, surveying 10 to 15 year old over three years. In this sense, Ševčíková and Šmahel (2009) found that of the older adolescents (16–19 years) 14.1% were in the role of the target, while of the younger adolescents (12–15 years) 7.6% experienced the target perspective. However, the situation was reversed for aggressors, as the higher proportion of aggressors was among 12–15 year old's (1.8%) rather than 16-to-19 years old's (1.2%). Mishna et al. (2010) reported an increased likelihood for older girls (grades 10 and 11) being exposed to cyberbullying compared to older boys, although this difference was not seen between girls and boys in lower grades (grades 6 and 7). Contrarily, while Ortega-Ruiz et al. (2009) indicate a significant peak in victimization at the age of 14, they also indicate that victimization decreased significantly between the ages of 12 and 17. Regarding cyberbullying, Schneider et al. (2012) report similar findings, with this phenomenon decreasing slightly from 9th grade to 12th grade (from 17.2 to 13.4%). Likewise, Waasdorp et al. (2018) observed that compared to middle school students, high school students were less likely to be affected by the various forms of victimization with the exception of cyberbullying.

Age (school grade level) also seemed to moderate the association between cyberbullying victimization and students' engagement. While a positive association between cyberbullying victimization and emotional engagement was stronger for high school students than for middle school students, a negative association between cyberbullying victimization and cognitive-behavioral engagement was stronger for middle school students than for high school students (Yang et al., 2020).

Gender was also hypothesized to play a role in frequency and type of cyberbullying behaviors students encountered. Gradinger et al. (2009) observed that 8% of male students reported having sent mean text messages, e-mails, videos or photographs, while only 3% of female students reported doing so. Similarly, Baldry et al. (2019) observed that boys tended to be only bullies and bullies/victims, while girls tended to be uninvolved and only victims. These results are in line with those found by Ortega-Ruiz et al. (2009) who found that more females reported being victims of cyberbullying, both *via* mobile phone (6.3% females vs. 2.4% males) and *via* the Internet (9.1% females vs. 6% males) and Schneider et al. (2012) who found that the victims of cyberbullying are more often girls than boys (11.1% vs. 7.6%). Contrarily, Low and Espelage (2013) found that female middle school students had higher levels of cyberbullying, with the extent decreasing over time. That girls are not only victims of cyberbullying, but also take on the role of perpetrators, was also shown by Mishna et al. (2010). Here, however, the form of cyberbullying was a relevant factor. According to this, boys were more likely to be victims or perpetrators of direct bullying (e.g., threatening), while girls were more likely to be victims or perpetrators of indirect bullying (e.g., spreading rumors). In this study 3% of participants believed they were bullied because of their gender and 1% indicated bullying others because of their target's gender. In addition, Mehari and Farrell (2018) observed that aggressive behaviors did not show different patterns of relations according to gender and Longobardi et al. (2020) found that gender had no significant effect on any of the variables in their study.

The ethnic background also seems to play a role in online victimization and harassment. At the first of three measurement time points, for example, Low and Espelage (2013) showed that African American youth reported higher levels of endorsement of cyberbullying compared to White participants. These findings are contrasted by other researchers, who found no significant differences in overall reporting of cyberbullying by race or ethnicity (Schneider et al., 2012). These results are in line with those found by Mishna et al. (2010), in which Canadian students who did not speak English at home were not at a higher risk of being bullied. In addition, no differences in the prevalence of cyberbullying were found when the language spoken at home was considered. However, it was found that students who spoke English at home were more likely to spread rumors online than students who did not speak English at home. In this study 6% of the participants believed they were bullied online because of their race, while 3% of participants reported online bullying because of the target's race. Concerning the role of race, Ybarra et al. (2011) assessed the relative likelihood of reporting experiences of violence, such as bullying victimization, where the minority race (Black/African American) was found to be protective of all victimization experiences.

#### Individual and Contextual Factors

Other predictors for cyberbullying included personal traits and attitudes. For instance, Ang and Goh (2010) documented a three-way interaction in which high affective empathy moderated the effects of low cognitive empathy on cyberbullying for girls compared to boys. With regard to potential risk and/or protective factors, Schultze-Krumbholz and Scheithauer (2009) also considered empathy in their study. They found that cyberbullies and cybervictims showed less empathy and higher levels of relational aggression compared to students who did not engage in cyberbullying.

Barlett et al. (2019) examined the relationship between positive cyberbullying attitudes and subsequent cyberbullying perpetration in a longitudinal study of 3,000 Singaporean adolescents over a three-year period. They found that children with stable high or increasingly positive attitudes toward cyberbullying behaviors were also more likely to engage in such acts.

Using Data from a school-based census of about 20,400 youth, Schneider et al. (2012) showed that cyberbullying is far more frequent among nonheterosexual youth (33.1%), compared to heterosexual youth (14.5%). In their study, Mishna et al. (2010) asked the more than 2,100 participants whether they thought sexuality led to their bullying (2%) or was the reason they bullied others (2%).

In their study, including data from more than 43,200 adolescents from middle and high schools, Waasdorp et al. (2018) showed that overweight youth were more likely to report being a victim of cyberbullying (obese youth had even a 66% higher risk of being victims of cyberbullying). The findings of their study are supported by the work of Mishna et al. (2010), in which more than one in ten (11%) felt they have been victims of cyberbullying because of their appearance. According to this study, other characteristics that participants felt led to their bullying were disability (2%), family (2%) and school performance (5%).

School performance was also part of the investigation in other studies, with particular respect to the relationship between lower school performance and online victimization. While Bossler et al. (2012) reported that lower school performance is a predictor of online victimization, in the study of Schneider et al. (2012) no causality relationship between these two factors was analyzed, thus considering cyberbullying victimization as a potential predictor for school performance and vice versa. Referring to school as an important place for adolescents, a lower school attachment was also found to increase the likelihood of victimization online (Schneider et al., 2012) and, contrary to expectations, the negative influences of cyberbullying victimization on cognitive-behavior engagement were actually enhanced when students perceived a positive school climate. At the same time, the positive relationship between cyberbullying victimization and emotional engagement was mitigated by perceptions of a positive school climate (Yang et al., 2020).

Considering cyberbullying from the perpetrator's point of view, again appearance seems to be an important factor. Thus, further in the study of Mishna et al. (2010) 6% of the perpetrators stated that the appearance of the victim was the reason for their attacks. According to this study, other characteristics that participants stated as a reason for bullying others were disability (1%), school performance (3%) and family (2%). That cyberbullying occurs between parties who are familiar with each other or would even consider each other friends could also be demonstrated by Mishna et al. (2010). Here, friends (52%) were the most frequent targets of cyberbullying behavior. The influence of friends was also highlighted by Bossler et al. (2012), who positively associated a higher percentage of friends misbehaving on the computer with victimization. Besides the situation with friends, factors that an adolescent faces at home, and especially the quality of the caregiver-child relationship, seem to influence the likelihood of cyberbullying (Ang and Goh, 2010). In this context, parental monitoring and also the use of protective software seem to be associated with higher levels of cyberbullying (Bossler et al., 2012; Low and Espelage, 2013). At the same time, however, general use of technology also appears to be an indicator of an increased likelihood of being exposed to and experiencing violent media (Ybarra et al., 2011).

#### Overlap Between Traditional Bullying and Cyberbullying

The overlap between traditional bullying and cyberbullying has been investigated in several studies included in this review, revealing both similarities and differences between the two phenomena. Students' tendency not to report cyberbullying and their reasons for doing so are consistent with findings from studies examining traditional bullying. According to Mishna et al. (2010), these reasons include fear of retaliation or that the bullying could get worse. However, that young people also fear losing Internet or cell phone privileges seems to be rather a concern that occurs only in the context of disclosing cyberbullying.

Looking at the victims' perspective, the different forms of bullying often seemed to occur in parallel. Mitchell et al. (2011), analyzing data from more than 2,000 adolescents ages 10 to 17, found that 96% of youth who experienced online victimization also reported offline victimization during the same time period. Here, the offline victimizations linked most closely to online victimization were sexual victimizations (e.g., sexual harassment) and psychological and emotional abuse. The findings are in line with those found by Gradinger et al. (2009), who also found that most of the cyberbullying victims were also victims of traditional bullying at the same time.

In contrast, other studies, such as by Schultze-Krumbholz and Scheithauer (2009), concluded that cyberbullying is more common compared to traditional bullying. That cyberbullying and traditional bullying differ in frequency was also shown by Ortega-Ruiz et al. (2009). According to their study, however, the two phenomena are inversely related: significantly more adolescents were targeted by traditional bullying (two in ten) than by cyberbullying (one in ten). One in five participants reported being affected by both types of bullying.

Likewise, the consequences for victims of both forms appear to have similarities. For example, Schneider et al. (2012) found that the level of distress was highest for victims of both cyberbullying and school bullying. Additionally, Ortega-Ruiz et al. (2009) observed similar emotional responses to cyberbullying *via* the Internet and indirect bullying as a special type of traditional bullying (e.g., threats or insults). Emotions cited by victims included anger, stress, or fear. Although Low and Espelage (2013) also mention that cyberbullying seems to have significant overlaps with non-physical bullying (i.e., verbal and relational bullying), longitudinal analyses showed, according to them, less overlap between the different forms.

A connection between bullying at school (in the sense of traditional bullying) and cyberbullying is also assumed by Ševčíková and Šmahel (2009), who hypothesize that the reason for this could be the non-anonymous relationship between perpetrators and victims.

### Predictors of Cyberhate

#### Socio-Demographic Variables

In their study involving more than 700 Finnish youth aged 15–18 who used Facebook as a social medium, Oksanen et al. (2014) analyzed the extent to which this age group is exposed to and victimized by online hate material. Two-thirds, and thus the majority, of the youths stated that they had already encountered online hate material, with 21% of the respondents having been victims of online hate material themselves. Furthermore, the authors observed that 70% of the participants more accidentally came across the online hate material, while 22% of the youth intentionally searched for this type of content. As a result of their analysis, they state that none of the sociodemographic variables (e.g., age, gender, living with parents) were found to be significant predictors of exposure to online hate material. Further, they link victimization by online hate material to various social and psychological factors, such as negative offline experiences.

These results are consistent with those found by Wachs et al. (2021), who reported neither significant differences in gender between girls (19%) and boys (15.4%) as related to cyberhate victimization, nor age differences between victims and non-victims of cyberhate.

Although socio-demographic data do not appear to be influential in the context of cyberhate, it is worth noting that, first, in the study by Oksanen et al. (2014) online hate material most frequently targeted at ethnicity/nationality (50%) and religious belief/faith (43%), and second, victims of online hate material were more likely to report material in which sex/gender was targeted compared to non-victims.

#### Individual and Contextual Factors

Study findings presented by Oksanen et al. (2014) suggest that certain states of agitation may increase the likelihood to be a victim of online hate material. For example, in contrast to those who did not perceive themselves as victims of online hate material, youth who did perceive themselves as victims were more likely to report being worried. This aspect was taken up by the research group in a later paper based on the same data set (Räsänen et al., 2016). In this, they concluded that worrying about becoming a victim of online hate material increases the likelihood of actually becoming a victim online.

According to the results of Oksanen et al. (2014), the hate material that victims saw online often directed at sexual orientation (68%), physical appearance (61%) and disability (31%). In addition, the authors observed that adolescents who were exposed to hate material online were more active online, not studying and their attachment to family was low. Specifically to the role played by being very active online, Ybarra et al. (2011) had achieved similar findings, and suggest that general technology use is an important factor in predicting risk for violent exposures (eg, hate sites) and experiences online.

The role of the family was again addressed in the study by Räsänen et al. (2016), but the analysis of the effects of covariates in the proposed model excluded the possibility to prove a correlation between living with parents and cyberhate victimization (even though basic statistics showed that not living with parents doubled the risk of online victimization).

Moreover, the authors report that the likelihood of becoming a victim of hate online is higher among youth who visit harmful sites on the Internet, the likelihood increases for those who deliberately search for this type of content, and the odds of victimization are almost four times higher for those producing hate materials. In line with this, Pauwels and Schils (2016) found that measures of extremism through new social media are associated with self-reported political violence. This relationship is most pronounced when users actively search for extremist content, with self-reported political violence being linked to various offline associations (e.g., racist and delinquent peers).

Looking at individual and contextual factors, Wachs et al. (2020) analyzed the influence of family affluence on cyberhate victimization. As the results of their study show, no differences were found between victims of cyberhate with low family affluence (33.9%), middle family affluence (31.7%), and high family affluence (35%).

Finally, Räsänen et al. (2016) found that, in contrast to the hypothesis that the number of friends on Facebook would increase exposure to hate material, this factor seems not to increase the likelihood of victimization. Slightly different is the influence of friends on cyberhate exposure, since Oksanen et al. (2014) found that 8% of the interviewed adolescents encountered hate material *via* a link from a friend.

#### Overlap of Cyberhate With Other Forms of Aggressive Behavior

As Wachs et al. (2020) showed, adolescents use similar coping strategies for dealing with cyberhate as they do for dealing with cyberbullying. Therefore, the authors not only assume conceptual and empirical overlaps between the two phenomena, but also that both forms have a similar impact on adolescents' behavior and emotions.

Based on the results of their study involving 1,900 French students, Blaya et al. (2020) noted that percentages of young people involved as victims or perpetrators were much higher offline than online.

In addition, they found that as victimization at schools (offline behavior) increased, the likelihood of exposure to hate material online (online behavior) also increased. Furthermore, their findings indicate that students who insult or threaten others at school also spread hate messages against others online. The way cyberhate is related to other forms of aggressive behavior is shown by the further results of the study. Thus, a weaker relationship was observed between cyberhate victimization and cyberhate perpetration, a moderate relationship was observed between school bullying victimization and cyberhate perpetration, and a moderate relationship was observed between cyberhate victimization and school bullying perpetration.

That exposure to hate material can be associated with offline physical victimization was also noted by Oksanen et al. (2014). In their later work, Räsänen et al. (2016) again consider this factor, stating that the odds of online hate victimization are higher if the user has already experienced online victimization.

### Impact of Cyberbullying

Victimization through cyberbullying can result in very different effects. For example, some studies linked online victimization to psychological distress (Mitchell et al., 2011; Schneider et al., 2012), with the odds of distress appearing to remain constant over time (Ybarra et al., 2011). Other studies have shown that victims reported depressive symptoms (Ortega-Ruiz et al., 2009; Schneider et al., 2012; Low and Espelage, 2013), which, like somatic symptoms (e.g., bellyaches and stomach cramps), were most common when victims experienced both traditional bullying and cyberbullying compared with non-victims (Gradinger et al., 2009). According to Mitchell et al. (2011) greater trauma symptomatology, including depression in addition to anger and anxiety, was slightly but significantly linked to online victimization.

Stress is also a reaction that was observed associated with cyberbullying. For instance, Ortega-Ruiz et al. (2009) found that victims who were more affected by cyberbullying (both *via* the Internet and mobile phone) felt more stressed than occasional victims. In the case of cyberbullying *via* the Internet, more women (13.7%) than men (2%) reported feeling stressed. This finding is in line with the conclusion drawn by Baldry et al. (2019) who describe post-traumatic stress as a psychophysiological condition “resulting from stressful traumatic events such as school bullying and cyberbullying”.

In the context of cyberbullying, it was noted that some adolescents do not seem to be bothered by online attacks. This was shown, for example, in the studies by Ortega-Ruiz et al. (2009) and Mishna et al. (2010), the latter also finding a gender difference between males (55.6%) and females (28.6%), but only for cyberbullying *via* mobile phone. At the same time, however, various negative emotions have been reported that appear to be the result of cyberbullying. Across different studies, victims referred to feeling afraid and/or scared (Ortega-Ruiz et al., 2009; Mishna et al., 2010; Patchin and Hinduja, 2010). Others reported feeling alone, defenseless and worried, with females (30.6%) more likely than males (5.6%) to be worried by cyberbullying *via* cell phone (Ortega-Ruiz et al., 2009). Longobardi et al. (2020) observed a negative correlation of cyber victimization and perceived subjective happiness, which is consistent with the findings of Mishna et al. (2010), in which victims reported feeling sad. Feelings of embarrassment and upset (Ortega-Ruiz et al., 2009; Mishna et al., 2010) were also reported, with the number who felt very or extremely angry as a result of victimization remaining constant over a 36-month observation period (Ybarra et al., 2011). In addition, adolescents expressed feeling angry, as seen in the studies by Ortega-Ruiz et al. (2009) and Mishna et al. (2010), the latter group showing that more females (37%) than males (18%) reported feeling angry when bullied *via* the Internet.

As studies show, adolescents who experienced multiple forms of bullying and victimization appear to be at higher risk for poor adjustment. According to Gradinger et al. (2009), perpetrators who performed bullying online and offline were at highest risk for externalizing adjustment problems (e.g., reactive or instrumental aggression), whereas victims who experienced bullying online and offline were at highest risk for internalizing adjustment problems (e.g., depressive and somatic symptoms). In addition, adolescents who performed and experienced bullying online and offline were at highest risk for both externalizing and internalizing adjustment problems. In this context, it is worth mentioning the study by Waasdorp et al. (2018), which links victimization (including the experience of cyberbullying) to adjustment and social-emotional problems in addition to childhood obesity. And also the study by Mitchell et al. (2011) can be highlighted here, in which online victimization was strongly associated with delinquency (e.g., physically harming other children or adults, intentionally damaging things that belong to others, cheating on tests, skipping school) during the period studied.

Similarities between perpetrators and victims of cyberbullying do not only seem to exist with regard to adjustment problems. Thus, Patchin and Hinduja (2010) found a moderate relationship between both low self-esteem and cyberbullying offending and between low self-esteem and cyberbullying victimization. At the same time, however, victims of cyber- and/or school bullying are the ones who show an increased risk for suicidal behaviors. Zaborskis et al. (2018), analyzing data from a cross-national survey conducted in 2013 and 2014, showed that victims were at higher risk of suicidal thoughts, plans, and attempts regardless of the type of bullying they experienced. Among young people from Lithuania and Luxembourg (in addition, young people from Israel were interviewed), the association between cyberbullying and suicidal behavior was even greater compared to bullying that happened at school. Consistent with these findings Schneider et al. (2012) report suicide attempts of among victims of online and offline bullying, with cyberbullying victims (9.4%) more affected than school bullying victims (4.2%).

### Impact of Cyberhate

According to Oksanen et al. (2014), there is an inverse relationship between cyberhate victimization and general psychological well-being, with victims of hate material more likely to be unhappy. In the context of emotional health, which can thus be affected by cyberhate victimization, coping strategies emerge into focus. In their study, Wachs et al. (2020) addressed the question of how adolescents deal with cyberhate. Taking into account differences in gender, age, socioeconomic status, and victimization status of the youth, six different coping strategies were confirmed. To mitigate the negative effects of cyberhate, adolescents primarily used constructive coping strategies, namely Technical coping (i.e., blocking a person), Assertiveness (telling the person to stop), and Close support (distracting oneself by spending time with friends). According to the authors, the fact that young people responded in this way indicates high levels of digital literacy which they know how to use, as well as high levels of self-efficacy. The remaining three strategies included Helplessness/Self-blame (not knowing what to do), Retaliation (do it back), and Distal advice (go to the police). Considering gender and age, girls were more likely to use all coping strategies (except Retaliation), and younger adolescents were more likely to use Technical coping strategies than older adolescents. Socioeconomic status was relevant to the extent that Distal advice and Technical coping were more common among adolescents with lower socioeconomic status than among peers with higher socioeconomic status.

## Discussion

The papers selected for this review provide precise indications on the factors that characterize the phenomena of cyberbullying and cyberhate, both in terms of predictive variables and their impact on adolescents. As the focus of this review was on cyberbullying and cyberhate as two interlinked instances of cyber-aggression, we compare the predictors and effects of one or the other phenomenon, and show how many of these factors characterize both cyberbullying and cyberhate, thus highlighting the level of overlap between them.

We have decided to analyse the predicting variables and consequences of the two phenomena separately, instead of framing them under the broader umbrella of cyber-aggression. The comparison follows this analysis. In such a way, we have the possibility to catch a new perspective on the investigation of these two phenomena.

Before presenting the results of this comparison, it should also be pointed out that the selected articles do not always provide a whole picture of the individual factors, and for this reason it has been necessary to enrich the description of some of them with additional sources of literature.

### Overlapping Between Predictors of Cyberbullying and Cyberhate for Adolescents

Amongst the common factors that can predict perpetration and victimization in both cyberbullying and cyberhate, the role of the family has drawn the attention of many scholars. Specifically, the role of the family has been analyzed from different perspectives, sometimes favoring the aspects related to the emotional parent-child relationship, sometimes considering the family as a proxy of the social guardianship that can be a deterrent to cyber-aggression. The latter is particularly present in papers presenting studies that have borrowed the Routine Activity Theory (Cohen and Felson, 1979), one of the main theories of criminology, to explain the phenomena of cyber-aggression.

The importance of the parent-child relationship is highlighted both in the literature on cyberhate, with Oksanen et al. (2014) who found that weak attachment to family significantly predicts exposure to online hate content, and in the literature on cyberbullying, with Ang and Goh (2010) who highlighted the importance of positive caregiver-child relationships in reducing cyberbullying behavior among adolescents. These findings are consistent with similar studies on bullying and cyberbullying; among others, Murphy et al. (2017) found that attachment to parents is a deterrent to both becoming bullies and becoming victims of bullying, factors; Wang et al. (2009) found that bullying and cyber-bullying were similarly related to low parental support.

Surprisingly, the protective role of the families against the risks of cyberbullying, which is highlighted in numerous studies in the literature, including the impressive work by Li et al. (2021) with almost 215,000 school-aged children across 41 countries, has not been explicitly targeted in the papers on cyberbullying selected for this review, with the only exception of the paper by Low and Espelage (2013), who found a positive association between parental monitoring and higher levels of cyber-bullying perpetration (only for white adolescents). The guardianship offered by the parents against the risks of online hate exposure and victimization has been analyzed in Oksanen et al. (2014) and Räsänen et al. (2016). These studies did not find a correlation between family guardianship and online hate material exposure (Oksanen et al., 2014), nor was it possible to prove a correlation between living with parents and cyberhate victimization (Räsänen et al., 2016).

Although this would seem to indicate that the protective role of the family is a further overlapping factor between the phenomena of cyberbullying and cyberhate, as no significant link with either phenomenon was found in the literature reviewed, these results should be further commented on in light of the fact that they seem to contradict numerous studies on cyber-aggression (Li et al., 2021). Of particular significance, Räsänen et al. state in their paper: “Therefore, simply living with one's parents does not appear to ensure guardianship. Thus, it is difficult to interpret the lack of significance of this variable” (p. 14).

A possible interpretation could be found by investigating the mode and quality of guardianships exercised by parents. In this regard, it is worth mentioning the recent study by Wachs et al. (2021) that has furtherly analyzed the relationships between cyberhate victimization and the form of parental mediation, and found that instructive parental mediation is negatively associated with cyberhate victimization, while restrictive parental mediation determines the opposite effect. This confirms, moreover, the results of a previous study on the protective role of the family (Papatraianou et al., 2014), where the authors highlight the importance of instructive parental mediation: “Strong family relationships within the context of a young person's home can also help young people overcome online adversity, along with family permissions to use technology in a safe way”.

Closely related to the role of adolescent-family relations on cyberbullying and cyberhate, and with similar outcomes, is the role played by relations with friends. The literature analyzed does not provide consistent results, nor is it possible to give an unambiguous interpretation by extending the literature analysis to articles not included in this review. Specifically, Mishna et al. (2010) found that friends are the most frequent targets of cyberbullying attacks (in 52% of the cases studied), and this percentage increases to 84% in an earlier study by Ybarra and Mitchell (2004). Although less evident, the negative role of friends is also confirmed in relation to cyberhate, where Oksanen et al. (2014) found that friends are one of the sources from which adolescents receive links to hate material (in 8% of the cases analyzed), thus favoring their exposure to cyberhate. If these results were generalisable, it could be assumed that as the number of friends increases, the risks of Cyberbullying victimization or cyberhate exposure and victimization should increase. However, Räsänen et al. (2016) found that an increase in the number of friends on Facebook did not correspond to an increase in the risk of cyberhate exposure; the same result was reached by Kaakinen et al. (2018). Similarly, Wang et al. (2009) found that having more friends is not associated with cyberbullying.

The synthesis of these results could be found by prioritizing the analysis of the quality of relationships with friends or of the behaviors usually carried out by friends, rather than focusing on the number of friends. Bossler et al. (2012) underline that friends who caused most online harassment were those who committed various forms of computer deviance. The quality of the relationship with friends in relation to the phenomena of cyberbullying is underlined by subsequent works (not selected for this review), which recognize the protective role of friends against cyber-aggression: Papatraianou et al. (2014) have pointed out how strong and supportive friend relationships can support female adolescents' resilience toward online risks and aggression; similarly, Zych et al. (2019) have verified that the quality of relationships with friends is a strong protective factor against cyberbullying. Nevertheless, other scholars have only partially confirmed these results. For example, Bedrosova et al. (2022), who analyzed these aspects with samples of adolescents in the Czech Republic, Poland and Slovakia, found that friendship support was negatively related to cyberhate in the Czech Republic and Poland, but not in Slovakia and, even more surprisingly, friendship support was negatively related to cyberbullying only in the Czech Republic. Similar results were found by Kaakinen et al. (2018) who analyzed, with samples of American, British, German and Finnish adolescents and young adults, how cognitive social capital in the offline context (i.e., trust and sense of belonging in a group of friends) influences cyberhate victimization. In addition to the finding that the number of Facebook friends was not associated with online hate victimization reported above, the authors found that trust and sense of belonging in a group of friends was negatively associated with online hate victimization in all samples, but not for the Finnish one. With everything considered, we therefore encourage further studies on the role of friends in relation to cyberbullying and cyberhate.

The constructs related to sexuality (sexual orientation; sexual identification; etc.) represent a further element of overlapping between cyberbullying and cyberhate, being predictors of perpetration, victimization and exposure to online hate material (Mishna et al., 2010; Schneider et al., 2012; Oksanen et al., 2014). This is not surprising, given that the sexual sphere has always been a reason for discrimination, both at an individual level and with regard to groups that feel the need to unite in order to fight against discriminatory stereotypes that societies cannot ignore (Russell et al., 2001; Robin et al., 2002; Williams et al., 2003).

### Overlapping Between the Impact of Cyberbullying and Cyberhate on Adolescents

The dimension that offers the major number of insights on the overlap between the consequences of cyberbullying and cyberhate on adolescents' individual well-being and emotions. In fact, the negative effects of cyber-aggression on emotional perception was found by authors who analyzed overall subjective happiness, direct emotional responses to experiences and long term emotional states induced by cyberbullying victimization and perpetration, as well as by cyberhate victimization or exposure (Ortega-Ruiz et al., 2009; Mishna et al., 2010; Ybarra et al., 2011; Longobardi et al., 2020; Wachs et al., 2020). In particular, this confirms Wachs et al. (2021) argument that the impact of cyberhate and cyberbullying on adolescents' emotions may be similar. Specifically to this point, it is worth mentioning Catherine Blaya, one of the authors of the EU report on the relation between cyberhate and kids (Machackova et al., 2020), who points out that “the emotional consequences are significant not only for victims but also for witnesses even though they are not targeted by the posted hateful contents. Both groups report experiencing anger and hate following their exposure or victimization” (as reported in Bedrosova, 2020). This confirms that the boundary between exposure to cyberhate and cyberhate victimization regarding its impact on users' emotions is extremely blurred (Machackova et al., 2020).

Strictly related to the effects of cyberbullying and cyberhate on adolescents' emotions, many studies have reported negative effects of cyberbullying victimization and perpetration on individuals' wellbeing, mainly consisting in depressive symptoms, somatic symptoms, post-traumatic stress symptoms and psychological distress (Gradinger et al., 2009; Ortega-Ruiz et al., 2009; Mitchell et al., 2011; Schneider et al., 2012; Fales et al., 2018). Although the selected literature does not provide similar information for adolescents who were exposed to or victims of cyberhate, an online survey administered to 1,512 adolescents (13–18 years.) in 2016 in UK revealed that young people who had been exposed to online hate content reacted to it with anger (37%), sadness (34%) and shock (30%) feelings (UK Safer Internet Centre, 2016). A distinct study involving young people in six countries slightly older than adolescents (18–25 years.) achieved similar results; respondents who had been exposed to online hate speech content reported almost the same negative emotional feelings as the adolescents in the UK survey: anger, sadness and shame (Reichelmann et al., 2020). Hence, findings from both studies on the consequences of cyberhate exposure and victimization identified symptoms which are common to the ones reported by the literature on cyberbullying, thus highlighting a further area of overlapping between cyberbullying and cyberhate.

Adolescents' coping strategies for cyberhate have been analyzed by Wachs et al. (2020), while the literature on cyberbullying selected for this review does not adress the issue of how adolescents deal with cyberbullying attacks. Nevertheless, the overlap has been highlighted by Wachs et al., who found out that adolescents use similar coping strategies for dealing with cyberhate as they do for dealing with cyberbullying. Specifically, the conclusions achieved by the authors are similar to those pointed out by other authors who have studied adolescents' coping strategies for cyberbullying (Livingstone et al., 2011; Machackova et al., 2013; Sticca et al., 2015).

Papers in this review underline the role of online activities in cyberbullying and cyberhate phenomena. As expected, the more adolescents spend their time online, the more they are involved in cyberbullying and cyberhate exposure (Ybarra et al., 2011; Oksanen et al., 2014; Räsänen et al., 2016). In this sense, the frequency in using Internet online tools is a predictor for both cyberbullying and cyberhate experiences. Ybarra et al. (2011) extend this concept, confirming that technology use in general is a predictor of both cyberbullying experiences and cyberhate exposure.

### Cyberbullying and Cyberhate: Distinguishing Features

The analysis of the literature shows that the concepts of cyberbullying and cyberhate are in part overlapping, but have some characteristics that distinguish them from each other. In particular, by examining the results related to adjustment problems and the ideation of suicide, some important differences can be observed.

Adjustment problems and suicide have been targeted by some of the papers on cyberbullying selected for this review. Specifically, Waasdorp et al. (2018) found that adolescents who have been victims of cyberbullying appear to be more likely to experience adjustment problems; Gradinger et al. (2009) revealed that both bullies and victims are at high risk of adjustment problems, especially if they are involved in both face-to-face and cyberbullying experiences; Schneider et al. (2012) and Zaborskis et al. (2018) identified attempted suicide as a consequence of cyberbullying.

These themes are not present in the literature on cyberhate, probably because they reflect a deep psychological discomfort that can lead to extreme gestures such as suicide, a discomfort that emerges when the victim of the attack is the individual adolescent rather than a group of people (even if the adolescent identifies with the group). Previous research on the consequences of discrimination (online and offline) on adolescents' mental well-being can support this assertion. Discrimination is, in fact, a transversal theme to cyberbullying and cyberhate, where in the first case it is a tool aimed at hurting the individual, while in the second case it is a manifestation of hatred against a group of individuals (based on gender, race, religion, etc.), with which the adolescent may or may not recognize himself. Studies analyzing the effects of discrimination against individuals confirm how the risk of adjustment problems and the number of suicidal ideations and attempts increase for adolescents who have been discriminated against (Sinclair et al., 2012). The results are different if the discrimination is directed at a group. Particularly relevant is the work of Tynes et al. (2008) who present the results of a cross-sectional survey with 264 US high school students aged 14–18 years old to examine the impact of online racial discrimination on adolescents' psychological well-being. The authors distinguish between individual and vicarious discrimination: the former includes acts of discrimination that are explicitly directed at the individual, similar to what occurs in cyberbullying. Vicariuos discrimination refers to discrimination acts directed at same-race adults and peers in the adolescent's life, similar to what occurs in cyberhate. This study confirmed that individual racial discrimination is significantly related to depression and anxiety. However, vicarious discrimination does not correlate with measures of psychological adjustment, thus confirming our assertion that attacks on groups of individuals typical of cyberhate are experienced less dramatically by adolescents than attacks experienced by cyberbullying victims.

Another aspect that differentiates the two types of cyber-aggression analyzed in this review is that certain individual and personal characteristics often related to physical appearance (obesity, overweight, disability) can be predictors of cyberbullying victimization (Mishna et al., 2010; Waasdorp et al., 2018), but are not present among the predictors of cyberhate victimization. This is inherent in the defining characteristics of the two phenomena, since cyberbullying is an aggressive behavior against a person, whereas cyberhate is against a group of individuals, and therefore individual and personal physical factors have much less relevance. Exceptions are those physical traits that can be associated with ethnic groups (e.g., skin color; eye shape, etc.) and are often used as the basis for discriminatory phenomena. This would seem to include the physical appearance which Oksanen et al. (2014) include among the predictors of cyberhate victimization, although they do not specify which specific traits of physical appearance they refer to.

The analysis of the literature has shown that gender is a predictor for cyberbullying perpetration and victimization (Gradinger et al., 2009; Ortega-Ruiz et al., 2009; Mishna et al., 2010; Schneider et al., 2012; Low and Espelage, 2013), with a few exceptions (Mehari and Farrell, 2018; Longobardi et al., 2020). In contrast, gender does not appear to be a predictor for cyberhate exposure and victimization (Oksanen et al., 2014; Räsänen et al., 2016; Wachs et al., 2020). This result seems surprising in light of the fact that the gender is one of the categories targeted by haters (such as race, religion, etc.), but at the same time it confirms the lack of consistent findings in the literature on the relationships between gender and cyberhate perpetration, exposure and victimization (Bauman et al., 2021).

Similarly, it was not possible to determine an overlap between cyberbullying and cyberhate with regard to race/ethnicity, cultural context, language spoken at home (here considered as a proxy of ethnicity). In fact, the literature on cyberhate clearly indicates that race is a predictor of cyberhate exposure and victimization (Oksanen et al., 2014), as could be expected, since race appears as one of the discriminating factors that lead haters to attack groups of people on the basis of the color of their skin, their ethnicity, and their culture. On the contrary, the analysis of the selected literature (Mishna et al., 2010; Ybarra et al., 2011; Schneider et al., 2012; Low and Espelage, 2013) does not clearly show a causal link between race and cyberbullying experiences. This confirms what has already been pointed out in previous studies. In particular, in a previous review in which the relationships between cyberbullying, race/ethnicity and mental health outcomes were analyzed, the authors indicated that young whites are bullied more than their non-white peers, but clarify that it is not possible to establish whether this is a direct relationship (consequence of race and ethnicity), or rather is due to other factors that differentiate youth of color and their white peers in terms of technology ownership, social media preferences, and socioeconomic backgrounds (Edwards et al., 2016).

Finally, the impact of self-esteem, empathy and high levels of relational aggression on cyberhate experiences has not been sufficiently analyzed in the literature, so that it is not possible to make comparisons with what emerged for cyberbullying (Schultze-Krumbholz and Scheithauer, 2009; Ang and Goh, 2010; Patchin and Hinduja, 2010).

## Conclusions

The first and most evident results from this review are that the cyberhate issue related to adolescents is less investigated than cyberbullying, and most of the papers dealing with one or the other phenomenon lacks of a holistic perspective, rooted in the broader concept of cyber-aggression, which makes it possible to approach cyberbullying and cyberhate as two distinct but often interconnected phenomena. In particular, the literature on cyberbullying lacks references to cyberhate, whereas the papers on cyberhate sometimes refer to literature on cyberbullying.

Nevertheless, by comparing the predictors and outcomes of cyberbullying and cyberhate, important overlapping factors between the two concepts emerge.

The most evident overlapping factors, as highlighted by this review, are the importance of the parent-child relationship to reduce the risk of cyber-aggression; the constructs related to sexuality (sexual orientation; sexual identification; etc.) as predictors of both phenomena; the protective role of the families against cyberbullying and cyberhate attacks, provided that parents offer instructive mediation while restrictive parental mediation determines the opposite effect; the role of good quality friendship relationships as deterrent against cyberbullying and cyberhate attacks; the impact of cyberbullying and cyberhate on adolescents' emotions as well as their consequences on individuals' wellbeing, mainly consisting in depressive symptoms, somatic symptoms, post-traumatic stress symptoms and psychological distress; the same coping strategies put in practice by victims of the two phenomena.

In addition to the factors common to cyberbullying and cyberhate, the literature highlights some of the characteristics that distinguish each of the two phenomena. In particular, differences concern the adjustment problems and the ideation of suicide, which have been found in studies on cyberbullying but not on cyberhate; individual and personal characteristics, often related to physical appearance (obesity, overweight, disability), as predictors of cyberbullying victimization only; the gender as a predictor for cyberbullying perpetration and victimization, while it does not appear to be a predictor for cyberhate exposure and victimization; the lack of a well-defined overlap between cyberbullying and cyberhate with regard to race/ethnicity, cultural context, language spoken at home (here considered as a proxy of ethnicity); the impact of self-esteem, empathy and high levels of relational aggression on cyber-aggression, even though this issue has not been sufficiently analyzed in the literature on cyberhate.

We argue that the results of this review can stimulate future research on cyberbullying and cyberhate where the two phenomena are analyzed as two interlinked instances of cyber-aggression, while respecting their distinctive features. Moreover, further research should investigate the effectiveness of prevention and intervention programs based on the shared commonalities and reciprocal influence of cyberbullying and cyberhate (e.g., the same coping strategies should be assessed against their capacity to empower adolescents regarding cyberhate and cyberbullying), according to a holistic approach to the general problem of cyber-aggression in adolescence.

## Data Availability Statement

The original contributions presented in the study are included in the article/supplementary material, further inquiries can be directed to the corresponding author/s.

## Author Contributions

GF: conceptualization, formal analysis, and paper drafting and revising. DT: conceptualization and paper drafting and revising. LS: conceptualization, formal analysis, data curation, methodology, and paper drafting. VS: formal analysis, data curation, methodology, and paper drafting and revising. SE: conceptualization and paper revising. All authors contributed to the article and approved the submitted version.

## Funding

This work has been developed in the framework of the project COURAGE—A social media companion safeguarding and educating students (No. 95567), funded by the Volkswagen Foundation in the topic Artificial Intelligence and the Society of the Future.

## Conflict of Interest

The authors declare that the research was conducted in the absence of any commercial or financial relationships that could be construed as a potential conflict of interest.

## Publisher's Note

All claims expressed in this article are solely those of the authors and do not necessarily represent those of their affiliated organizations, or those of the publisher, the editors and the reviewers. Any product that may be evaluated in this article, or claim that may be made by its manufacturer, is not guaranteed or endorsed by the publisher.

## Footnotes

^1^ https://datareportal.com/reports/digital-2020-july-global-statshot

^2^ https://datareportal.com/reports/digital-2021-april-global-statshot

^3^ https://www.aacap.org/AACAP/Families_and_Youth/Facts_for_Families/FFF-Guide/Social-Media-and-Teens-100.aspx

^4^https://ec.europa.eu/eurostat/web/products-eurostat-news/-/edn-20210630-1)

^5^ https://www.cdc.gov/violenceprevention/youthviolence/bullyingresearch/fastfact.html
